# Morphology and Functional Anatomy of the Recurrent Laryngeal Nerve with Extralaryngeal Terminal Bifurcation

**DOI:** 10.1155/2016/9503170

**Published:** 2016-07-14

**Authors:** Fuat Cetin, Emin Gürleyik, Sami Dogan

**Affiliations:** Department of Surgery, Düzce University Medical Faculty, 81650 Düzce, Turkey

## Abstract

Anatomical variations of the recurrent laryngeal nerve (RLN), such as an extralaryngeal terminal bifurcation (ETB), threaten the safety of thyroid surgery. Besides the morphology of the nerve branches, intraoperative evaluation of their functional anatomy may be useful to preserve motor activity. We exposed 67 RLNs in 36 patients. The main trunk, bifurcation point, and terminal branches of bifid nerves were macroscopically determined and exposed during thyroid surgery. The functional anatomy of the nerve branches was evaluated by intraoperative nerve monitoring (IONM). Forty-six RLNs with an ETB were intraoperatively exposed. The bifurcation point was located along the prearterial, arterial, and postarterial segments in 11%, 39%, and 50% of bifid RLNs, respectively. Motor activity was determined in all anterior branches. The functional anatomy of terminal branches detected motor activity in 4 (8.7%) posterior branches of 46 bifid RLNs. The motor activity in posterior branches created a wave amplitude at 25–69% of that in the corresponding anterior branches. The functional anatomy of bifid RLNs demonstrated that anterior branches always contained motor fibres while posterior branches seldom contained motor fibres. The motor activity of the posterior branch was weaker than that of the anterior branch. IONM may help to differentiate between motor and sensory functions of nerve branches. The morphology and functional anatomy of all nerve branches must be preserved to ensure a safer surgery.

## 1. Introduction

Both the anatomical integrity and motor activity of the recurrent laryngeal nerve (RLN) must be preserved during thyroid surgery for a complication-free operation. The RLN has many anatomical variations that complicate thyroid surgery; moreover, full exposure of the cervical part of the RLN is mandatory to avoid surgical iatrogenic injury to the nerve. Full anatomical knowledge, including all RLN variations, is required for its proper identification and exposure. Extralaryngeal terminal bifurcation (ETB) of the nerve is a common variation, which makes dissection of the nerve branches difficult. ETB has a mean incidence of occurrence of approximately 30% and may occur bilaterally in 25% of patients with bifurcated RLNs. The incidence of larger extralaryngeal branches of the RLN has been reported between 18% and 42% in many surgical series [1–7]. On the other hand, this incidence was reported up to 65% in surgical series and even up to 92% in anatomic studies on cadavers including thin branches from RLN to adjacent structures [8–11].

Besides the morphological anatomy of the RLN, the functional anatomy is of paramount importance for the proper action of the laryngeal musculature. Anatomical integrity does not always guarantee motor activity of the nerve. Therefore, an intraoperative assessment of the functional anatomy of the nerve contributes significantly to the exposure of a morphologically intact RLN. The motor activity of nerve branches can be assessed by intraoperative nerve monitoring (IONM) and is a widely accepted adjunct to anatomical identification of the RLN [12–16].

In the present study, we aimed to establish the morphology of terminal branches in bifurcated RLNs and to evaluate their functional anatomy by IONM.

## 2. Materials and Methods

This prospective study included 36 patients who had an RLN with an ETB. RLNs were identified and exposed until the laryngeal entry point during thyroid surgery. Terminal branches of the RLN were macroscopically determined and exposed throughout the cervical courses. The functional anatomy of the terminal branches was evaluated by means of IONM.

### 2.1. RLN Dissection Technique

After medial mobilization of the bilateral lobes of the thyroid gland, the RLN was identified and fully isolated using a conventional lateral approach. The nerve was carefully exposed to the laryngeal entry point. If a macroscopically and clearly delineated ETB was identified along its cervical course, location of the bifurcation point on the cervical part of the nerve was determined.

### 2.2. Extralaryngeal Terminal Bifurcation of the RLN

Division of the RLN occurred along its cervical course prior to laryngeal entry. Similar or closely sized branches were macroscopically observed intraoperatively. These cervical branches enter separately into the larynx. Location of the bifurcation point on the nerve segment along its cervical course was classified according to previous surgical classification as follows [2]:

  Arterial, where bifurcation occurs at or adjacent to the crossing of the RLN and ITA.
  Postarterial, where bifurcation occurs on the distal nerve segment between the RLN-ITA crossing and laryngeal entry.
  Prearterial, where early bifurcation occurs on the proximal nerve segment before the RLN-ITA crossing.

### 2.3. Intraoperative Neuromonitoring of the RLN

We performed IONM to determine the functional anatomy of the terminal branches of the bifurcated nerve. IONM was performed using the Nerve Integrity Monitor (NIM-Response 3.0 System; Medtronic Xomed, Jacksonville, FL, USA). The nerve branches were stimulated after complete exposure under direct vision, which provided conduction of stimulating electricity until an innervated musculature. IONM was performed as a four-step procedure on RLNs with ETB [17]:

  V1: vagus nerve (VN) stimulation before the identification of the RLN.
  R1: RLN stimulation when first identified at the tracheoesophageal groove.
  R2: stimulation of the main RLN trunk before bifurcation after complete dissection of the lateral thyroid lobe, including
    R2a, stimulation of the anterior branch of the RLN,
    R2b, stimulation of the posterior branch of the RLN.
  V2: VN stimulation after complete dissection of the lateral thyroid lobe.

Intraoperatively, the sound signal of motor electrophysiological activity was obtained from the device while the wave amplitude was measured and recorded. The sound signal and electronic wave amplitude (as *μ*V) represented the proper functional anatomy of nerve branches.

Location of the bifurcation point on the nerve was determined after complete exposition of the RLN. The surgical anatomy of the bifurcated RLN was established by surgical dissection, and exposure of the cervical course of the nerve and the functional anatomy were assessed by IONM.

## 3. Results

During the study period, 46 RLNs with an ETB were determined in 36 patients (31 total thyroidectomies and one right and four left hemithyroidectomies). Thirty (83.3%) of our patients were female. The average age was 51.8 years (range: 27–70 years). ETB was bilateral in 10 of the 31 total thyroidectomy cases. We studied the morphology and function of 46 RLNs with an ETB (Table 1).

**Figure 1:**
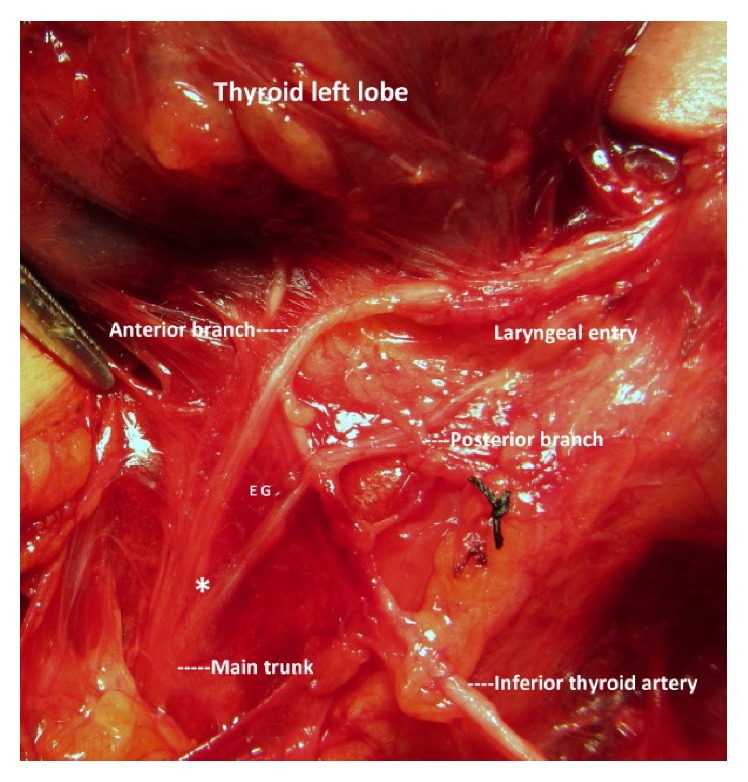
Prearterial bifurcation of the left RLN; early division before nerve-artery crossing. ^*∗*^Bifurcation point.

**Figure 2:**
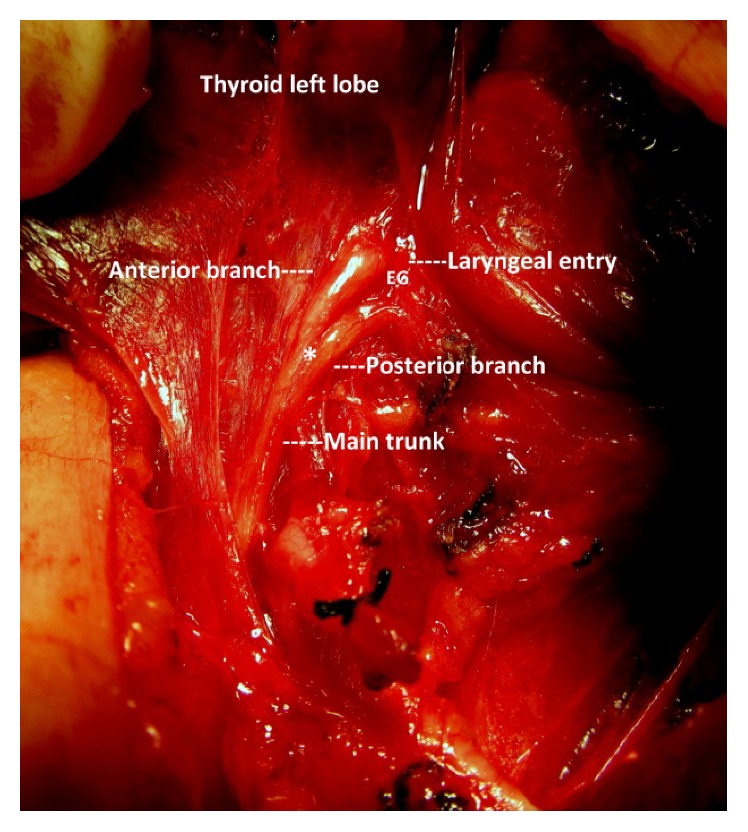
Postarterial bifurcation of the left RLN; late division distal to nerve-artery crossing. ^*∗*^Bifurcation point.

In half of the bifid nerves, the location of the bifurcation point on the nerve along its cervical course was observed at a distal segment between the RLN-ITA crossing and laryngeal entry point (Table 2). After full exposure, bifurcation points were observed at different segments of the RLN along its cervical course (Figures 1 and 2).

The functional anatomy and motor activity of neural tissue were evaluated in 46 RLNs with an ETB. A positive sound signal of motor activity was obtained from all anterior branches of the bifurcated RLNs. Four (8.7%) posterior branches also produced a positive signal after electrophysiological stimulation (Table 3). The electrical conductivity power of nerve branches was measured by wave amplitude after application of the stimulator probe. Motor activity in four posterior branches produced wave amplitudes 25–69% of those produced in the corresponding anterior branches of the RLNs (Table 4).

## 4. Discussion

Identification and exposure of the cervical segment of the RLN are mandatory during thyroid surgery. Surgeons must preserve a both morphologically and functionally intact nerve for a safe thyroidectomy. On the other hand, the RLN has many anatomical variations that compromise the safety of surgery. Nonrecurrent course of the right nerve is a rare variation [18–20]. The RLN has various relations with the Berry ligament, inferior thyroidal artery, and Zuckerkandl's tubercle [21–26]. Another anatomical variation of the RLN is the ETB along its cervical course prior to the laryngeal entry. The incidence of an RLN with an ETB has been reported to be 25–45% in exposed nerves during thyroid surgery. A bilateral ETB also occurs in a considerable number of patients [2–5, 11, 27]. IONM is a useful tool for evaluating the motor function of laryngeal nerves during thyroid surgery. Nerve integrity monitoring is an important adjunct to visually identify the RLN and determine its intact motor activity upon completion of a thyroidectomy. We used IONM to establish the functional anatomy of RLNs in both anterior and posterior branches of a nerve with an ETB.

Sometimes, thyroid surgeon may observe terminal branches of the RLN prior to laryngeal entry. In the case of an ETB, we must separately expose larger terminal branches to prevent injury to the nerve branches. Based on previous studies, we can comment that ETB is a common anatomical variation [2, 5, 6, 11, 28]. Locating the division point is crucial to securely identify and expose the neural structures and protect the integrity of the nerve. Besides the common occurrence of ETBs, variable locations of the bifurcation point complicate the exposure of the nerve. Our results demonstrated that bifurcation of the RLN occurred at different segments of the nerve. Early division, before the nerve-artery crossing, has been observed in 11% of bifid nerves. Surgeons must be extremely cautious while exposing the RLN to prevent injury to the extralaryngeal branches; moreover, they should be aware of the various locations of a bifurcation point on different nerve segments. In the majority of patients, the division point is located between the ITA crossing and laryngeal artery [2, 17, 28]. An ETB can be a potential cause of injury due to visual misidentification as this variation cannot be predicted preoperatively and may be associated with a higher rate of nerve injury. The injury prevalence has been reported as 5.2% and 1.6% for bifid and nonbifid nerves, respectively [5]. Knowledge of such a variability will help to visually identify the RLN and thereby decrease complication rates and increase the safety of thyroid surgery.

While the morphological integrity of an RLN is required for an uncomplicated surgery, it does not always ensure proper motor activity. In the case of bifid RLNs, the location of motor fibres in the nerve branches is extremely important for the preservation of motor function. Based on our results, the motor activity in all anterior branches showed that these branches provided motor innervations of the laryngeal musculature. The anterior branches of all bifurcated nerves contain motor fibres, in accordance with the findings of previous reports confirming that 100% of anterior branches are pathways of motor activity [4–6, 17, 29]. On the other hand, posterior branches also contain motor fibres and uncommonly conduct motor stimulation to the larynx. In our present study, the rate of motor function in the posterior branches was 8.7% while two recent papers reported rates of 1.3% and 8% [5, 29]. We believe that the most dangerous situation is misidentification and misinterpretation of the relatively larger posterior branch as the main trunk of the nerve. In this situation, the anterior branch is under the greatest risk and the inadvertent division of motor fibres may lead to laryngeal muscle palsy, despite the surgeon believing that the nerve was preserved. In this situation, assessing the motor function of the nerve by IONM may help surgeons to securely identify the main trunk and terminal branches of the RLN. Both intermittent and continuous nerve monitoring are a safe, effective, successful, and reliable method for evaluating the functional anatomy of the RLN as an adjunct to visual identification, particularly in cases of anatomical variations [30–32]. Anatomical variations of the nerve, including an ETB, may be considered high risk situations. Besides visual identification, functional identification of the nerve by IONM may be extremely helpful for establishing its morphological and functional anatomy and for preventing injury to bifurcated RLNs.

In the case of a bifid RLN, the motor activity in the main trunk must be checked before the bifurcation and afterwards in both branches. Comparisons of wave amplitudes between the anterior and posterior branches of the RLN provided useful information about the power of their conductivity. The motor activity of posterior branches had considerably lower amplitudes than those of the corresponding anterior branches. These results revealed that the density of motor fibres in the posterior branches was less than that in the anterior branches. We found a limited number of publications regarding a comparison of motor activity between branches of bifurcated RLNs [5, 17, 29]. The posterior cricoarytenoid (PCA) muscles are the only abductors (respiratory) in the laryngeal muscle group that in some cases receive motor fibres from the posterior branch of the RLN. Less than half of the PCA muscles contain any type of nerve branches from the posterior division [33]. The clinical reflection of injury to the posterior branches with motor activity cannot be predicted due to the variable motor fibre content in these branches. Therefore, the severity of vocal and/or respiratory impairment is also unpredictable and will undoubtedly differ among such patients, and the surgeon must preserve the morphological and physiological integrity of all nerve branches.

A common anatomical variation of the RLN is an ETB prior to the laryngeal entry. The anterior branches always contain motor fibres while the posterior branches seldom contain motor fibres. The posterior branch has a weaker motor activity than the anterior branch. Injury to motor nerve branches may impair vocal and/or respiratory function to variable degrees, although the density of motor fibres in the injured posterior branch may increase the severity of this impairment. The functional anatomy of a bifid nerve established by IONM may help to differentiate between motor and sensory branches. Based on the location of the motor fibres in all anterior and in some posterior branches, the morphology and functional anatomy of all neural structures must be preserved to ensure a safe and complication-free surgery.

## Figures and Tables

**Table 1:** Nerves at risk and recurrent laryngeal nerve (RLN) with extralaryngeal terminal bifurcation (ETB).

Patients with bifurcated RLNs		RLNs at risk	RLNs with ETB	Occurrence of ETB		Side of ETB	
				Bilateral	Unilateral	Right	Left
Total thyroidectomy	31	62	41		21	7	14
				10		10	10
Right hemithyroidectomy	1	1	1	Ø	1	1	Ø
Left hemithyroidectomy	4	4	4	Ø	4	Ø	4

Total	36	67	46	10	26	18	28

**Table 2:** Location of bifurcation point on the nerve segments along the cervical course of the recurrent laryngeal nerve.

RLN^*∗*^ segments	Right RLN	Left RLN	Total
	*N* = 18	*N* = 28	*N* = 46
Arterial	7 (38.9)^*∗∗*^	11 (39.3)	18 (39.1)
Postarterial	10 (55.6)	13 (46.4)	23 (50)
Prearterial	1 (5.5)	4 (14.3)	5 (10.9)

Total	18 (100)	28 (100)	46 (100)

^*∗*^RLN: recurrent laryngeal nerve; ITA: inferior thyroidal artery.

^*∗∗*^Numbers in parentheses are percentages.

**Table 3:** Functional anatomy of posterior branches based on sound signal of motor activity.

Posterior branches of bifurcated RLNs	Bifid NaR^*∗*^	Signal positive (motor activity)	Signal negative (sensitive branch)
		(%)	(%)
Right RLNs posterior branches	18	3 (16.7)	15 (83.3)
Left RLNs posterior branches	28	1 (3.6)	27 (96.4)

Total posterior branches	46	4 (8.7)	42 (91.3)

^*∗*^NaR: nerve at risk.

**Table 4:** Wave amplitude (*µ*V) in anterior and posterior branches with motor activity.

Nerve branches	Case 1	Case 2	Case 3	Case 4
Anterior (*µ*V)	967	2140	1259	1882
Posterior (*µ*V)	244	627	571	1302
Rate	25.2%	29.3%	45.4%	69.2%
